# Supplementary material for: Discovery and application of insertion-deletion (INDEL) polymorphisms for QTL mapping of early life-history traits in Atlantic salmon
Source: BMC Genomics. 2010 Mar 8;11:156. doi: 10.1186/1471-2164-11-156 (PMC2838853; doi:10.1186/1471-2164-11-156)
Supplement: Additional file 2 — Information on developed 76 locus single-run INDEL panel in Atlantic salmon. Information on fluorescence labeling, primer concentrations, PCR pooling and links to alignments, INDEL motifs and GENESCAN (Burge and Karlin 1997) predictions of genes/exons are available in html format. [file 1471-2164-11-156-S2.ZIP › Additionalfile2/snpsummary7444.html]

```
Cluster 2188 Contig 1

prev  Summary    Contig List  next
```

Size of Consensus sequence = 1210

Number of sequences = 22

Minimum redundancy = 6

Key

A gi|117866428|gb|EG939124.1|EG939124 EST\_ssal\_evf\_22780 ssalevf mixed\_tissue Salmo salar cDNA Salmo salar cDNA clone ssal\_evf\_530\_002\_rev 5', mRNA sequence  
B gi|117846954|gb|EG919650.1|EG919650 EST\_ssal\_evf\_58582 ssalevf mixed\_tissue Salmo salar cDNA Salmo salar cDNA clone ssal\_evf\_578\_275\_rev 5', mRNA sequence  
C gi|117870468|gb|EG943164.1|EG943164 EST\_ssal\_evf\_26416 ssalevf mixed\_tissue Salmo salar cDNA Salmo salar cDNA clone ssal\_evf\_534\_378\_rev 5', mRNA sequence  
D gi|117526305|gb|EG858032.1|EG858032 EST\_ssal\_eve\_48370 ssaleve thyroid Salmo salar cDNA Salmo salar cDNA clone ssal\_eve\_565\_317\_rev 5', mRNA sequence  
E gi|117857539|gb|EG930235.1|EG930235 EST\_ssal\_evf\_32369 ssalevf mixed\_tissue Salmo salar cDNA Salmo salar cDNA clone ssal\_evf\_543\_005\_rev 5', mRNA sequence  
F gi|117457692|gb|EG789911.1|EG789911 EST\_ssal\_evd\_53527 ssalevd thymus Salmo salar cDNA Salmo salar cDNA clone ssal\_evd\_572\_054\_rev 5', mRNA sequence  
G gi|24384564|gb|CA054321.1|CA054321 ssalrgb523021 mixed\_tissue Salmo salar cDNA, mRNA sequence  
H gi|117490998|gb|EG823215.1|EG823215 EST\_ssal\_evd\_24874 ssalevd thymus Salmo salar cDNA Salmo salar cDNA clone ssal\_evd\_532\_131\_rev 5', mRNA sequence  
I gi|117534199|gb|EG865644.1|EG865644 EST\_ssal\_eve\_27271 ssaleve thyroid Salmo salar cDNA Salmo salar cDNA clone ssal\_eve\_537\_028\_rev 5', mRNA sequence  
J gi|117840246|gb|EG912942.1|EG912942 EST\_ssal\_evf\_14805 ssalevf mixed\_tissue Salmo salar cDNA Salmo salar cDNA clone ssal\_evf\_518\_120\_rev 5', mRNA sequence  
K gi|117840244|gb|EG912940.1|EG912940 EST\_ssal\_evf\_14804 ssalevf mixed\_tissue Salmo salar cDNA Salmo salar cDNA clone ssal\_evf\_518\_120\_fwd 3', mRNA sequence  
L gi|85158796|gb|DW589934.1|DW589934 sbra1-013AD03.y1\_#0575.2 DIAS\_SBRA Salmo salar cDNA 5', mRNA sequence  
M gi|117534198|gb|EG865643.1|EG865643 EST\_ssal\_eve\_27270 ssaleve thyroid Salmo salar cDNA Salmo salar cDNA clone ssal\_eve\_537\_028\_fwd 3', mRNA sequence  
N gi|117490997|gb|EG823214.1|EG823214 EST\_ssal\_evd\_24873 ssalevd thymus Salmo salar cDNA Salmo salar cDNA clone ssal\_evd\_532\_131\_fwd 3', mRNA sequence  
O gi|117457681|gb|EG789900.1|EG789900 EST\_ssal\_evd\_53526 ssalevd thymus Salmo salar cDNA Salmo salar cDNA clone ssal\_evd\_572\_054\_fwd 3', mRNA sequence  
P gi|117870469|gb|EG943165.1|EG943165 EST\_ssal\_evf\_26417 ssalevf mixed\_tissue Salmo salar cDNA Salmo salar cDNA clone ssal\_evf\_534\_378\_fwd 3', mRNA sequence  
Q gi|117846955|gb|EG919651.1|EG919651 EST\_ssal\_evf\_58583 ssalevf mixed\_tissue Salmo salar cDNA Salmo salar cDNA clone ssal\_evf\_578\_275\_fwd 3', mRNA sequence  
R gi|117853514|gb|EG926210.1|EG926210 EST\_ssal\_evf\_28745 ssalevf mixed\_tissue Salmo salar cDNA Salmo salar cDNA clone ssal\_evf\_538\_045\_fwd 3', mRNA sequence  
S gi|117866427|gb|EG939123.1|EG939123 EST\_ssal\_evf\_22779 ssalevf mixed\_tissue Salmo salar cDNA Salmo salar cDNA clone ssal\_evf\_530\_002\_fwd 3', mRNA sequence  
T gi|117526293|gb|EG858020.1|EG858020 EST\_ssal\_eve\_48369 ssaleve thyroid Salmo salar cDNA Salmo salar cDNA clone ssal\_eve\_565\_317\_fwd 3', mRNA sequence  
U gi|57122885|gb|CX354326.1|CX354326 ssalrgb523021\_rev\_0 mixed\_tissue Salmo salar cDNA, mRNA sequence  
V gi|117857540|gb|EG930236.1|EG930236 EST\_ssal\_evf\_32370 ssalevf mixed\_tissue Salmo salar cDNA Salmo salar cDNA clone ssal\_evf\_543\_005\_fwd 3', mRNA sequence

4 SNPs detected

A B C D E F G H I J K L M N O P Q R S T U V  cosegregation weighted

518 . C C G C C G G G C . . G G C C . . . G G .   1/4 17.05
572 . . C . C C C - - C . C - - C C - . . - C C   3/4 54.55
573 . . T . T T T - - T . T - - T T - . . - T T   3/4 54.55
574 . . G . G G G - - G . G - - G G - . . - G G   3/4 54.55
